# EXAMINING THE ROLE OF OBJECTIVE AND SUBJECTIVE NEIGHBORHOOD CHARACTERISTICS ON HEALTH AND WELL-BEING IN MID-LIFE

**DOI:** 10.1093/geroni/igac059.2301

**Published:** 2022-12-20

**Authors:** Omar Staben, Frank Infurna, Kevin Grimm

**Affiliations:** RAND Corporation, Tempe, Arizona, United States; Arizona State University, Phoenix, Arizona, United States; Arizona State University, Phoenix, Arizona, United States

## Abstract

There is a long-standing literature that has documented how one’s neighborhood context has the potential to shape mental and physical health across the adult lifespan. An abundance of research documents how various components of the neighborhood, such as disorder, cohesion, in addition to pollution and sidewalk quality are linked to short- and long-term mental and physical health outcomes across adulthood. One key component that has been less studied within this literature is the extent to which objective or subjective neighborhood indicators exert a more potent impact on mental and physical health. Up to this point, much of the research has focused on subjective indicators of neighborhood context. This study explores whether and to what extent objective neighborhood factors of income inequality, residential stability, and greenness and subjective neighborhood factors of social ties, collective efficacy, and neighborhood disorder are predictive of mental and physical health in midlife. We use data from a sample of participants in midlife (n=800, aged 40-65) to analyze our research questions. Structural equation models found that both subjective and objective indicators of neighborhood were related to health and well-being when considered separately. When considered simultaneously, subjective neighborhood indicators (sense of community) mediated the association between objective constructs and health. Our findings demonstrate the independent associations between objective and subjective neighborhood context and highlight the particularly strong association between subjective context and health and well-being. Our discussion elaborates on how our findings can inform interventions and sparks future research aimed at exploring potential mechanisms underlying the associations found.

